# Resuscitation of Those Apparently Drowned

**Published:** 1888-09

**Authors:** 


### RESUSCITATION OF THOSE APPARENTLY DROWNED.

Every season greater or lesser number of persons are drowned at the summer watering-places. Imprudence in bathing or careless boating are the almost invariable causes of such deplorable accidents. Not infrequently the victims of such accidents are rescued before life is extinct, and could be resuscitated if the proper measures were resorted to in a prompt and effectual manner. In order, however, to accomplish results at once so urgent and desirable, every person should understand the few plain and practical rules that are usually relied upon to bring about restoration. These rules, as will be seen, are simple and can be employed by almost any one who can remember them and retains the self possession to apply them in an intelligent manner.

When a person drowns he suffers death from suffocation. Air has ceased to enter the lungs, and in place thereof the air passage and cells are filled with water. This is especially the case if a person breathes or gasps after sinking, in which event the water is sucked in and the air is forced out. If a person sinks and the body is recovered in five, ten, or fifteen minutes—perhaps even more—there are three natural conditions to be re-established as rapidly as possible: breathing, warmth, and circulation. The instant the body is in hand, begin the work for life, but let everything be done in a cool and methodical manner.

1. Loosen constricting clothing, Turn the person face downwards, then, bending over, clasp your arms under the lower portion of the person's breast and lift up and continue so doing for two or three seconds. This procedure will make the head lower than the rest of the body, at the same time it compresses the lower portion of the chest, thus tending to force the water out of the air cells. This process should be repeated two or three times after brief intervals. Don't hang the person up by the heels, roll him or her on a barrel, or do any useless and brutal acts.

2. Then turn the person upon the back, and if there is any dry clothing at hand, quickly tear off the wet garments and wrap with those that are dry and warm—for warmth is one of the essential conditions on which life depends.

3. Now commence the "Sylvester Method" for the restoration of respiration. This method is probably as good as any and has the advantage of being very simple.

"Place the patient on the back on nearly a level surface. Raise and support the head and shoulders on a small, firm cushion or folded articles of dress placed under the shoulder blades. Draw out the tongue and hold with a cloth of some kind in order to avoid its slipping back and preventing the entrance of air into the lungs. Now begin the imitation of breathing by kneeling at the patient's head and grasping his arms just above the elbows. Carry the arms steadily upwards from the body to above the head, and keep them stretched upwards for about two seconds. By this means air is drawn into the lungs. Then turn down the victim's arms, and press them gently and firmly for about two seconds against the side of the chest. By this means air is pressed out of the lungs. Repeat the measures alternately, deliberately, and perseveringly, about fifteen times in a minute, until a spontaneous effort to respire is perceived. Immediately upon which, cease to imitate the movements of breathing and proceed to favor the circulation and warmth."

4. Warmth is best promoted by removing the wet garments from the victim and replacing them with woolen blankets if these can be had. If they can not be had, use any covering at hand, providing it is warm and dry. Also employ, if it can be had, artificial warmth in the form of hot flannels and bottles filled with hot water. Friction, such as rubbing the patient with the bare hand or with flannels, also aids towards restoring warmth and exciting the circulation. Let it be borne in mind that warmth is one of the indispensable conditions of life, and it is ever one of the chief agents in restoring those who are apparently drowned.

5. The restoration of the circulation is the third object to be kept in view. Here warmth performs another important part, for it tends to relax the capillaries which are in a state of contraction from the effect of cold. It renders it possible for the blood to circulate, and it relieves the congested state of the internal organs. Rubbing not only aids in increasing heat but also favors greatly the motion of the blood. The efficiency of rubbing is increased by using such stimulents as turpentine, whiskey or salt water. Rubbing should be made towards the heart.

6. Finally, less important efforts, but still more worthy to be employed, are the cautiously passing of ammonia or hartshorn under the victim's nose ; allowing plenty of fresh air ; sprinkling cold water in the face ; "flipping" or slapping the face with the end of a towel wet with cold water ; keeping the victim flat on his back so as to favor the heart's action. As soon as there is ability to swallow, stimulents may be guardedly used, but they are of doubtful utility.

In conclusion there are four cardinal maxims to be borne in mind when attempting to restore those who are apparently drowned ; act promptly ; don't get excited ; use common sense ; persevere.

